# Impact of No-Clamping Partial Nephrectomy on Early Estimated Glomerular Filtration Rate Preservation

**DOI:** 10.3390/jcm13185491

**Published:** 2024-09-16

**Authors:** Piotr Falkowski, Maciej Jaromin, Miłosz Ojdana, Piotr Kutwin, Tomasz Konecki

**Affiliations:** 1st Urology Clinic, University Clinical Hospital No. 2 of the Medical University of Lodz., 90-419 Lodz, Poland; pfalkowski1@gmail.com (P.F.); miloszojdana77@gmail.com (M.O.); piotr.kutwin@umed.lodz.pl (P.K.); tomasz.konecki@umed.lodz.pl (T.K.)

**Keywords:** patrial nephrectomy (PN), laparoscopy, renal cell cancer (RCC), eGFR, off-clamp, on-clamp, AKI, urologic oncology

## Abstract

Incidences of kidney cancers are steadily increasing. The surgical resection of renal tumors remains the treatment of choice, and different techniques provide similar oncological outcomes. Minimally invasive methods, especially partial nephrectomy (PN), have emerged as the preferred method of tumor resection, both in traditional and robot-assisted laparoscopy. PN may be performed as an open or laparoscopic operation. On-clamp PN is a variant of PN that includes the clamping of renal vessels; off-clamp PN is performed without any ischemia. **Objectives**: To assess the short-term loss of eGFR after on-clamp and off-clamp PN. **Methods**: Data from 2021 to 2024 were retrospectively collected from a hospital database. The patients included in the study had a diagnosed kidney tumor that was confirmed by MRI or CT imaging. The patients were divided into two groups depending on the type of treatment they received: on-clamp PN or off-clamp PN. Hematocrit (HCT), hemoglobin (Hb) and eGFR were measured and compared. **Results**: Both groups had comparable preoperative HTC, Hb, and eGFR. eGFR loss 24 h after the procedure was 35.4% lower in the off-clamp group compared to the on-clamp group (*p* = 0.027). **Conclusions**: Off-clamp PN is a safe and viable method for kidney tumor resection, both in traditional and robot-assisted laparoscopy. This technique results in a smaller perioperative loss of eGFR, which relates to better short-term functional outcomes than on-clamp PN.

## 1. Introduction

### 1.1. Epidemiology

Renal cell cancer (RCC) contributes to 5–6% of overall cases of cancer in Western societies, with males being at higher risk of developing this type of cancer. According to predictions for 2024 in the United States, there will be 52,380 new cases of renal cancer in men and 29,230 new cases of renal cancer in women, with overall mortality around 17%. For comparison, in 2020, there were 76,975 new cases overall, with an overall mortality rate of 22% [1,2].

A population-based study from the United States provided insight into the epidemiology of renal cell cancer by analyzing 418,526 cases from 2000 to 2019. The risk of developing RCC is higher in men (63.41%) than in women (36.59%), and most cases were diagnosed in the 55–69-year-old age group (41.85%) [3].

### 1.2. Types of RCC

Renal cell cancers are divided according to histological types. The most common type is clear cell renal cell carcinoma (ccRCC), representing 70–75% of all renal cell cancers. ccRCC originates in the epithelium of proximal convoluted tubules in the renal cortex. It can be recognized in contrast-enhanced computed tomography using contrast uptake that is visible in the 120–180 HU range, with possible spots of necrosis.

Papillary renal cell carcinoma is diagnosed in 10–20% of all RCC cases. Papillary carcinoma originates in the epithelium of distal convoluted tubules. It is surrounded by a pseudo-capsule and is usually homogenous in contrast-enhanced CT imaging, sometimes imitating a Bosniak IIF- or III-type cyst.

Chromophobe renal cell carcinoma represents around 5% of all RCCs. It originates from intercalated cells of collecting ducts in a manner similar to that of oncocytomas. In contrast-enhanced CT imaging, chromophobe RCC appears more homogenous than ccRCC and is less vascular. Nevertheless, some chromophobe renal cell carcinomas may imitate ccRCCs. Other rare renal cell carcinomas include collecting duct RCC, medullary RCC, papillary adenoma, mucinous tubular RCC, and spindle cell RCC, among others.

Renal angiomyolipoma (AML) is a benign tumor that is usually spotted incidentally in abdominal USG or CT imaging. The prevalence of AML was assessed in a study analyzing abdominal ultrasounds of 61,389 patients from 1999 to 2009. AML was detected in 0.44% of the cohort, more often in women than in men [4]. Differentiation of AML is based on detecting a hypodense area (<10 HU) of fat deposits inside the tumor. Although AML is rarely symptomatic, it is not always possible to distinguish it from RCC since they may share similarities in both CT and MRI imaging [5].

Renal oncocytoma is another benign neoplasm, representing 3–7% of renal tumors. A typical sign of oncocytoma is a tumor with a central scar surrounded by a homogenous mass. Unfortunately, due to the heterogeneity of RCC in imaging, it is often not possible to distinguish oncocytomas from malignant tumors; therefore, tumors presenting as oncocytomas should be surgically resected [6].

Overall, renal cell cancers are not easy to distinguish in CT imaging and have many similarities—the final diagnosis is always dependent on the histological assessment of the specimen [7,8].

### 1.3. Surgical Treatment

Following trends from all over the world, minimally invasive procedures are overshadowing open surgeries and are clearly preferred by patients and clinicians alike. In the case of radical nephrectomy, minimally invasive surgery has several advantages over open surgery, namely, less blood loss during the procedure, a shorter hospitalization time, lower rates of readmission, and better overall survival and mortality rates. Postoperative complications were similar in both groups [9,10]. Similarly, when comparing open and laparoscopic PN, blood loss was lower with the laparoscopic approach, but oncological safety was comparable. Open PN was associated with a higher risk of acute kidney injury and a decrease in short-term renal function [11,12,13,14]. In our clinical experience, the laparoscopic approach is far superior to the open approach, not only regarding measurable clinical parameters but also quality and comfort of postoperative life. However, open surgery is often the only choice for resection of the biggest tumors or metastatic masses, for which laparoscopy is simply impossible. For this reason alone, open surgery is and shall always remain an important part of urology.

The off-clamp method is a variation of the laparoscopic PN that excludes clamping of the renal vessels from the procedure. The clamping of renal vessels induces ischemia in the operated kidney; the time of ischemia is related to the risk of acute kidney injury [14,15]. Omitting clamping of the renal vessel prevents ischemia and may be preferred in patients with renal vessel anomalies; as a tradeoff, it may lead to higher blood loss and much more difficult conditions during operations. A multi-institutional, randomized clinical trial that included 249 patients who qualified for PN (123 on-clamp, 126 off-clamp) reported the differences between those methods. Tumor size, gender, age, and base renal function were similar in both groups. Short-term renal function, blood loss, and complication rates were similar; positive margins were less prevalent in the off-clamp group (8.5% to 3.5%), although it was not statistically significant (*p* = 0.1). A systematic review of robot-assisted on- and off-clamp PN confirmed those findings, also reporting fewer positive margins in the off-clamp group (*p* = 0.02) [16,17]. A retrospective analysis of 63 patients monitored 15-year oncological and functional outcomes. After 15 years, renal function impairment was diagnosed in 14% of patients, recurrence in 2% and metastasis in 17%. The cancer-specific survival rate was 90%, and the overall survival rate was 72% (the median age of patients was 64 years on the date of operation) [18].

A reliable and affordable method of enhancing perioperative outcomes is the employment of intraoperative ultrasound (IOUS). Ultrasound is an easy and affordable imaging method and grants additional insight into the boundaries of the tumor and the depth of resection. A retrospective analysis compared the results of the utilization of IOUS during partial nephrectomy. The results showed shorter operating times, and reduced blood loss and ischemia time in the IOUS group [19]. Similar benefits of the application of intraoperative IOUS are true for robotic-assisted partial nephrectomy [20].

### 1.4. Functional Outcomes

Regardless of resection technique, kidney tumor operations negatively impact overall renal function. A meta-analysis that included 13,269 patients evaluated perioperative outcomes after partial and radical nephrectomy in the treatment of T1 RCC. PN provided significantly better GFR preservation 24 h after the surgery in comparison to radical nephrectomy (mean difference, 11.28; *p* < 0.0001) [21]. A study by Tanaka et al. evaluated functional outcomes after PN in 428 cases of RCC (mean tumor size: 3.5 cm). The preserved parenchymal mass of the operated kidney was 84%, and the preserved overall GFR was 89%. Postoperative functional outcomes depended mostly on the preoperative clinical status of the patient and the remaining renal parenchyma [22]. Interestingly, the renorrhaphy technique also has an impact on the preservation of GRF; single-layer suturing provides better functional outcomes than double-layer suturing [23].

## 2. Aim of the Study

The purpose of the study is to assess short-term functional outcomes after off-clamp PN operations and compare them with the results after on-clamp PN.

## 3. Materials and Methods

All PN procedures performed in our clinic from 2021 to 2024 were included in the study, excluding operations that included the removal of simple renal cysts. The decision to perform on-clamp or off-clamp PN depended on patients’ clinical status, TNM staging of the tumor, location of the tumor, and findings in diagnostic imaging (e.g., additional renal vessels); ultimately, the decision was at the discretion of the surgeon. All procedures were performed via the traditional (non-robot-assisted) 3D laparoscopic transperitoneal approach. There were no conversions to hand-assisted laparoscopy or open surgery.

Data were acquired retrospectively from the hospital’s database and operative reports. All operations were performed in the 1st Urology Clinic of the Military Medical Academy Memorial Teaching Hospital in Łódź, Poland.

Statistical analysis was performed using R Studio software (version 2023.12.1+402). Analyzed data included surgical margins and histopathological diagnosis, levels of hemoglobin (Hb), hematocrit (HCT), and eGFR before and 24 h after the procedure. Mean and median values for age and BMI were calculated for both on-clamp and off-clamp patient groups. A Shapiro–Wilk test was performed for tumor size, hemoglobin, hematocrit, and eGFR to check for normal distribution. Then, the Wilcoxon rank sum test with a continuity correction was performed to compare data between the on-clamp and off-clamp groups. Statistical significance of the differences in positive surgical margins was calculated with Fisher’s exact test.

## 4. Results

A summary of the features of the cohort is presented in Table 1. Preoperative characteristics of both groups were compared using a *t*-test or a Wilcoxon test; corresponding *p*-values are provided in each row. In both groups, the mean preoperative eGFR was below 90, but after adjusting for age, this value was in a normal, healthy range. Age, BMI, and preoperative Hb and HCT levels were similar in both groups. Patients who qualified for the off-clamp PN had, on average, a lower eGFR before the procedure than those who qualified for the on-clamp PN, but this difference was not statistically significant (*p* = 0.46). Tumor size was smaller in the off-clamp group, which was confirmed by a Wilcoxon test (*p* < 0.001); a Chi-square test of independence confirmed that the tumor sizes differed regarding distribution (*p* < 0.001). Perioperative transfusion and dialysis represented all instances of intervention during hospitalization after the procedure. The only incident of blood transfusion occurred in an 87-year-old patient on the third day after the procedure. The patient was provided with two units of packed red blood cells and two units of fresh, frozen plasma packs. The patient was discharged after further observation. In both groups, there were no incidences of AKI or renal replacement therapy after the procedures.

Table 2 presents a summary of histological cancer types in both groups with eventual positive surgical margins. Clear cell RCC was diagnosed in 59% of all cases (64% in the on-clamp group; 33% in the off-clamp group). Papillary RCC was diagnosed in 15.3% of all specimens (16.7% in the on-clamp group; 8.3% in the off-clamp group), and chromophobe RCC in 4.1%. Oncocytoma contributed to 7% of all cases (5% in the on-clamp group; 16.7% in the off-clamp group); AML contributed to 8.3% of all cases (4.2% in the on-clamp group; 29.2% in the off-clamp group). Due to the high disparity in the number of tumor types, the Wilcoxon test was invalid, and *p*-values were not provided.

**Figure 1:**
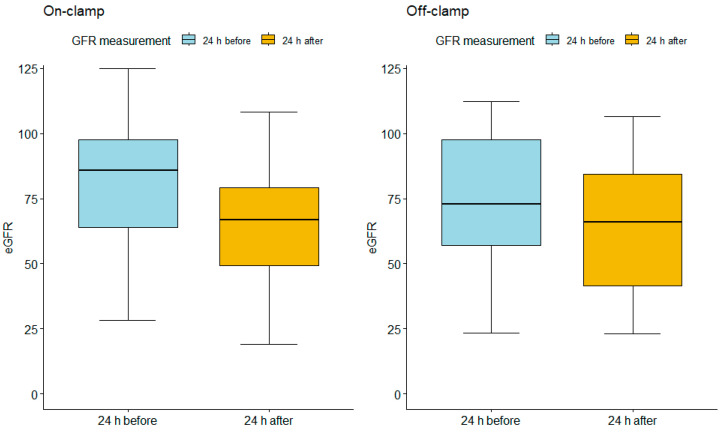
Box plots for on-clamp and off-clamp eGFR measurements. On-clamp 24 h before PN median (mean) = 85.9 (80.67), 95% CI = 38.87 113.00; 24 h after PN median (mean) = 66.8 (65.61), 95% CI = 24.13 104.62. Off-clamp 24 h before PN median (mean) = 73 (75.48), 95% CI = 32.02 111.82; 24 h after PN median (mean) = 65.9 (65.75), 95% CI = 27.19 106.37.

Measurements of short-term eGFR loss (Figure 1) were performed based on serum creatine levels measured 24 h before the operation and 24 h after the operation. For the on-clamp group, the average perioperative change in eGFR before and after PN amounted to 15.06 (mean eGFR = 80.67, 95% CI = 38.875–113.000; mean eGFR = 65.61, 95% CI = 24.1325–104.6175, respectively). For the off-clamp group, the average change in eGFR before and after PN amounted to 9.74 (mean eGFR = 75.49, 95% CI = 32.02–111.82; mean eGFR = 65.75, 95% CI = 24.1325–104.6175, respectively).

The changes from both groups were compared using the Wilcoxon test. The average loss of eGFR in the off-clamp group compared to the average loss of eGFR in the on-clamp group was lower by 35.4% (*p* = 0.027).

**Figure 2:**
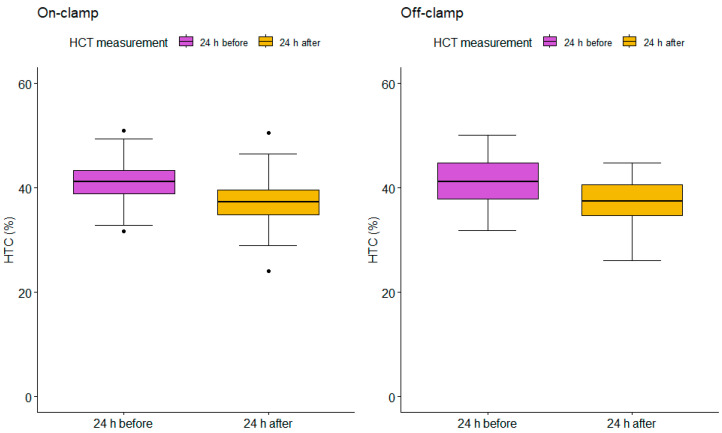
Box plots for on-clamp and off-clamp HCT (%) measurements. On-clamp 24 h before PN median (mean) = 41.20 (41.43), 95% CI = 38.87 113.00; 24 h after PN median (mean) = 37.30 (37.20), 95% CI = 24.13 104.62. Off-clamp 24 h before PN median (mean) = 41.15 (41.44), 95% CI = 33.5825 48.5475; 24 h after PN median (mean) = 37.40 (37.40), 95% CI = 27.19 106.37.

**Figure 3:**
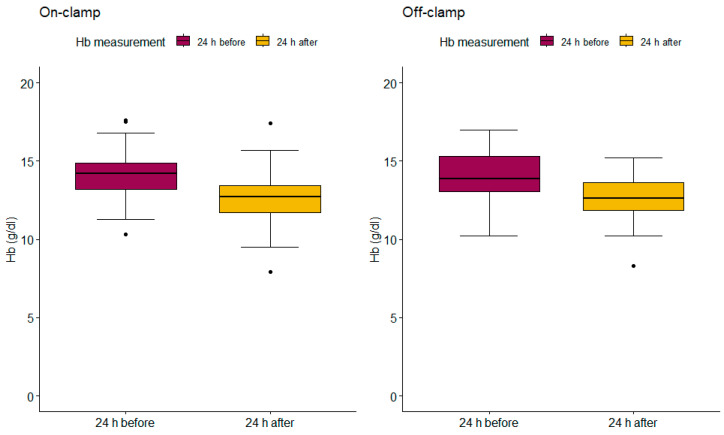
Box plots for on-clamp and off-clamp Hb (g/dL) measurements. On-clamp 24 h before PN median (mean) = 14.2 (14.14), 95% CI = 11.795 16.605; 24 h after PN median (mean) = 12.7 (12.57), 95% CI = 9.9975 15.1100. Off-clamp 24 h before PN median (mean) = 13.85 (14.07), 95% CI = 11.0625 16.7125; 24 h after PN median (mean) = 12.6 (12.60), 95% CI = 9.3925 15.0850.

Levels of HTC (Figure 2) and Hb (Figure 3) were measured using eGFR 24 h before and after PN. The on-clamp and off-clamp groups had remarkably similar outcomes regarding preoperative HTC levels (mean HTC = 41.43%; mean HTC = 41.44, respectively) and postoperative HTC levels (mean HTC = 37.20%; mean HTC = 37.40, respectively). Similarly, in the on-clamp and off-clamp groups, preoperative (mean Hb = 14.14; mean Hb = 14.07, respectively) and postoperative Hb levels (mean Hb = 12.57; mean Hb = 12.60, respectively) were comparable. Perioperative changes in HTC (*p* = 0.70) and Hb levels (*p* = 0.56) were not statistically significant.

## 5. Discussion

The off-clamp technique is becoming increasingly popular in robot-assisted and traditional laparoscopy; the safety and efficacy of this method have been proven in oncological and perioperative outcomes [18,24,25]. However, data on functional outcomes are scarcer, and the results are not unanimous. A multicenter RCT CLOCK study compared the absolute variation in estimated glomerular filtration rate (AV-GFR) after 6, 12, 18, and 24 months in patients after off-clamp (n = 164) and on-clamp (n = 160) PN. The median warm ischemia time oscillated between 14 and 15 min, and RENAL scores for tumors were <11 in all patients. The results show no statistically significant difference in AV-GFR, regardless of time after the procedure [26]. By contrast, a comparative study including 629 patients assessed the risk of developing chronic kidney disease (stage ≥ 3b) in an 8-year period after on-clamp and off-clamp PN; perioperative and oncological outcomes were also measured. The results of both the univariable and multivariable Cox regression analyses pointed to the on-clamp method as a predictor of developing chronic kidney disease (*p*-values of 0.036 and 0.005, respectively). In the on-clamp group, the risk of developing CKD in the 2-year period after surgery was 0.6%; this risk was 4.9% in the 5-year period after surgery and 15.5% in the 8-year period after surgery. In the off-clamp group, the risk of developing CKD was stable throughout the years and amounted to 0.8%. In summary, the on-clamp group had a 7.3-fold increase in risk of developing CKD. Perioperative complications, cancer-free survival, and overall survival were comparable between the two groups [27]. Such an increase in risk is quite surprising; more notably, in the 2-year period after surgery, the risk of developing CKD was comparable between both groups. It is also not clear if the clamping of renal vessels (median warm ischemia time of 16.3 min) is solely responsible for functional loss of the kidney 8 years after the procedure. A similar comparative study reported a difference in GFR loss 6 months after on-clamp vs. off-clamp PN, but the results were statistically insignificant when warm ischemia time was limited to 30 min [28].

The evidence presented proves that off-clamp PN is a viable method of kidney tumor resection. Analyses of the literature regarding perioperative outcomes are not always fully objective since the success of every surgical procedure depends on the proficiency of the surgeon. A study by Yossepowitch et al. [29] reported on postoperative outcomes in patients with positive surgical margins after PN; data were gathered from Mayo Clinic and Memorial Sloan Kettering Cancer Center.

Surgical margins were present in 13/609 cases (2%) from the Mayo Clinic and in 64/771 cases (8%) from Memorial Sloan Kettering. Both hospitals are tertiary referral centers, but the disparity in positive surgical margins is 4-fold [29].

A similar study on predicting positive surgical margins in patients after robot-assisted PN was conducted in another tertiary referral center (Careggi Hospital). The results show positive surgical margins in 109/1611 (6.7%) cases; interestingly, the off-clamp method was regarded as a risk factor for positive surgical margins [30].

The replication of the presented and cited results may not be guaranteed, especially in clinics not accustomed to the method. The learning process for perfecting off-clamp NSS technique requires years of commitment and practice. A study by Ferriero et al. compared the perioperative results of robot-assisted off-clamp NSS between the expert surgeon and the training surgeon. The expert surgeon treated patients with larger tumors and worse clinical status; nonetheless, after the propensity score matching of patient groups, there were no statistically significant differences in perioperative outcomes [31]. A study by Zhang et al. reported on the learning curve of an experienced surgeon in performing robot-assisted off-clamp PN. The study included 50 cases. The results of a multidimensional cumulative sum analysis show a reduction in operative time, consultation time, and hospital stay after performing 24 surgeries. No positive surgical margins were reported; blood transfusion was needed in three patients [32].

Changes in perioperative eGFR clearly pointed to off-clamp PN as a more favorable procedure. Loss of eGFR in the on-clamp group was significant—24 h after the procedure, renal function dropped by approximately 19% on average. A greater reduction in eGFR in the on-clamp group most probably occurred due to ischemia, which causes hypoxia and a short-term loss of nephron function. This is especially important in patients with chronic kidney disease, since even short-term reductions in GFR may accelerate the progression of the disease and lead to complications; the same considerations are true for patients with a single kidney. The vast majority of patients included in the study were diagnosed with pT1 (pT1a > pT1b) tumors. While considering the functional outcomes of off-clamp PN, it is also important to recognize the limitations of the PN method in general. Tumors that invade the renal pelvis or perinephric tissues (pT3) are unviable for partial resection. PN in patients with pT2 tumors is possible in theory but very difficult in practice and is possible only in a fraction of cases. Most probably, the difference in GFR reductions between off-clamp and on-clamp PN is more extensive for resections of tumors >4 cm in diameter; unfortunately, in our cohort, there were not enough tumors of this size to provide any statistically significant results. Whether this postoperative “shock” increases the risk of a long-term decrease in kidney function remains unclear; more research (especially prospective studies) is required.

This study had a few limitations and differences between the on-clamp and off-clamp groups. The sizes of the groups are unequal; this poses a challenge for comparisons between the two groups and makes some statistical tests (the Chi-square test of independence and the Wilcoxon rank sum test with continuity correction) less accurate. The researchers deliberately chose not to regulate or influence the size of the groups; the goal of the study is to provide a report on the benefits of off-clamp PN in an unfiltered clinical environment. A prospective, randomized study should provide groups of comparable size and further expand our knowledge of the field. The difference in tumor size between groups is also explained by clinical reasoning: tumors with a diameter above 4 cm were mostly qualified for the on-clamp procedure to avoid unnecessary blood loss. Cancer cells do not provide any functional value for the kidney; therefore, the amount of excised tumor cells should not affect eGFR directly. The impact of size and mass of resected tumors during PN on postoperative kidney function is unclear and should be assessed in a multivariate logical regression analysis. Unfortunately, due to the size of the samples, this method was not applicable in this study. Reductions in eGFR are caused by ischemia, renorrhaphy, trauma during surgery, and resection of healthy renal cortex with surgical margins; all of those parameters were similar in both groups. A lower eGFR before the procedure in the off-clamp group results from preoperative qualification—there was a bias to perform off-clamp PN in patients with impaired renal function whenever possible to retain eGFR. Similarly, there was a tendency to select patients with tumors imaged with CT and MRI and classified as benign for the off-clamp PN. It was motivated by the fact that visual conditions and maneuverability are often limited during off-clamp PN, so the potential for surgical margins might be higher.

A prompt diagnosis in the setting of acute kidney injury (AKI) is paramount for patient care, especially during the perioperative period. AKI is defined by presence any of the following conditions: an increase in serum creatinine ≥0.3 mg/dL (≥26.5 μmol/L) within 48 h; an increase in serum creatinine ≥ 1.5 times baseline in the last 7 days; or a urinary output of <0.5 mL/kg/h for 6 h [33]. This definition does not include any specific kidney injury markers, which may provide quicker and more precise insight into kidney function. A recent study by Lacquaniti et al. assessed the predictive value of urinary insulin growth factor binding protein-7 (TIMP2*IGFBP7) and serum mid-regional pro-adrenomedullin (MR-proADM) in the early diagnosis of AKI in patients after cardiac surgery. The results showed that an increase in TIMP2*IGFBP7 4 h after the surgery is a reliable predictor of AKI (sensitivity, 83.9%; specificity, 73.8%), and an increase in MR-proADM is an indication of the potential occurrence and severity of postoperative sepsis [34]. The application of biomarkers in the diagnosis of AKI requires differentiation between the underlying causes. In patients with hepatorenal syndrome, cystatin C and urinary neutrophil gelatinase-associated lipocalin levels may contribute to diagnosis and treatment [35]. Levels of urinary C-C motif chemokine ligand 14 provide valuable information in patients with persistent stage 3 AKI [36]. Biomarkers of AKI after PN may be measured with the NephroCheck^®^ Test (San Diego, CA, USA); a single-institution prospective study reported promising results in the diagnosis of AKI using a NephroCheck urine test 10 h after the surgery [37]. More research is needed to establish universal recommendations for early AKI diagnosis.

Off-clamp PN seems to be the best surgical method for the resection of small renal masses because it yielded very good functional and oncological results. In our opinion, wider popularization and promotion of the off-clamp technique will greatly benefit urological patients. 

## Figures and Tables

**Table 1:** Characteristics of on-clamp and off-clamp PN groups.

Characteristic	On-Clamp PN Group *n* = 120	Off-Clamp PN Group *n* = 24	*p*-Value
Gender	M = 71, F = 49	M = 14, F = 10	
Mean age (SD)	63.38 (10.71)	62.79 (11.68)	*p* = 0.91
Mean BMI (SD)	27.68 (4.04)	28.79 (4.18)	*p* = 0.28
Median eGFR before the procedure	85.9 mL/min/1.73 m^2^	73 mL/min/1.73 m^2^	*p* = 0.46
Median Hb before the procedure	14.2 g/dL	13.85 g/dL	*p* = 0.86
Median HCT before the procedure	41.20%	41.15%	*p* = 0.99
Mean tumor size (SD)	3.31 cm (SD = 1.47 cm)	2.05 cm (SD = 1.21 cm)	*p* < 0.001
Warm ischemia time	17 min	0 min	*p* = 1
Blood loss	134.21 mL	134.58 mL	*p* = 0.97
Surgical margins (%)	11 (9.17%)	2 (8.3%)	*p* = 1
Perioperative transfusion	1	0	*p* = 1

**Table 2:** Presentation of tumor types in on-clamp and off-clamp groups.

Tumor Type	On-Clamp PN Group (Positive Margins)	Off-Clamp PN Group (Positive Margins)
Clear Cell RCC	78 (9)	8 (0)
Papillary RCC	20 (0)	2 (1)
Chromophobe RCC	6 (1)	0
Renal Oncocytoma	6 (1)	4 (1)
Angiomyolipoma	5 (0)	7 (0)
Adult Cystic Nephroma	1 (0)	0
Multilocular Cystic Renal Neoplasm of Low Malignant Potential	2 (0)	1 (0)
Unidentified	1 (0)	0
Follicular Adenoma	0 (0)	1 (0)
Pseudocyst	1 (0)	1 (0)

## Data Availability

The raw data supporting the conclusions of this article will be made available by the authors on request.
